# A generalized atomic system of *N*-level atom within the framework of ($N-1$) JCM interacting with one-mode field solved via supersymmetric approach

**DOI:** 10.1038/s41598-025-32623-5

**Published:** 2026-01-08

**Authors:** R. A. Zait

**Affiliations:** Mathematics Department, Faculty of Science, Minia University, Minia, Egypt

**Keywords:** Supersymmetric Quantum Mechanics, Jaynes-Cummings Model, Multi-photon Transitions, Kerr Medium, Quasi-probability distribution *Q*-function, Atomic Inversion, Mathematics and computing, Physics

## Abstract

We study a generalized atomic system of *N*-level atom within the framework of ($N-1$) JCM interacting with one-mode field in the presence of Kerr medium and multi-photon transitions. We solve this system via supersymmetric approach. We show that the system possesses supersymmetric structure and construct its supersymmetric generators and their properties and diagonalize its Hamiltonian using supersymmetric unitary transformation. The atom-field eigenfunctions and eigenvalues of the system are obtained in two different cases of the initial conditions of the atom and the field mode. The evolution of the quasi-probability distribution *Q*-function and the atomic inversion is investigated for one case of the initial conditions. Their graphical results are presented and discussed for three special cases of the level number *N*, namely, $N=3$ which represents the three-level $\Lambda$-type system, $N=4$ which represents the four-level *m*-configuration system, and $N=5$ which represents the five-level double $\Lambda$-type system. The influence of the detuning and Kerr medium parameters on the behavior of these nonclassical quantities is presented graphically and discussed and found that they have important effects. We end by discussion and conclusions and some features and comments.

## Introduction

In the field of quantum optics, the most common model that describes the matter-field interactions is the so-called Jaynes-Cummings model (JCM)^1–3^, in which the interaction of a single two-level atom with one-mode of a cavity field is studied under the rotating wave approximations (RWA), and found that it possesses various nonclassical effects. Since the emergence of this model, many extensions have been proposed in various ways, for example, considering multi-level atom^4–7^, multi-mode field^8,9^ as well as multi-photon transition^10^ and multi-atom^11–13^, considering intensity-dependent coupling^14–17^, the presence of Kerr medium^18,19^ and Stark shift^10,20^, ... etc.

The main task in studying matter-field atomic systems like JCM and its various generalization is to construct the wave functions corresponding to the Hamiltonian of the system. One of the most interesting way to do this is via supersymmetric unitary transformation method^21–23^. It was shown that the JCM possesses supersymmetric structure^21–23^, and the corresponding Hamiltonian is diagonalized through supersymmetric unitary transformation and therefore its eigenstates and eigenvalues are obtained in this way. This method depends on constructing the supersymmetric generators of the system and then by diagonalizing the Hamiltonian of the system, one can easily obtain the corresponding wave functions. Once the wave functions are obtained, one can investigate any quantum aspects of the system. Moreover, it was shown that various generalized JCM possess supersymmetric structure too and can be solved in this way^24–28^. Also, the *V*-type three-level atom interacting with a single-mode field via multi-photon transitions is studied^29^ and some quantum effects are investigated. A multi-photon interaction of two collectively two-level atoms with two-mode field in the presence of Kerr medium and intensity-dependent coupling is studied and the evolution of various quantum features is investigated^30^. Moreover, a four-level double *V*-type atom interacting with a single-mode field via multi-photon processes in the presence of Kerr medium is also studied and some quantum effects are investigated^31^.

The aim of this article is to introduce a generalized matter-field atomic system of *N*-level atom within the framework of ($N-1$) JCM interacting with one-mode field in the presence of both Kerr medium and multi-photon transitions. We show that this system possesses supersymmetric structure and solve it via supersymmetric unitary transformation and obtain the corresponding wave functions. We study the evolution of both the quasi-probability distribution *Q*-function and the atomic population inversion as two main aspects of the system.

The organization of this article is as follows. In the next section, we introduce the considered atomic system. In section Supersymmetric generators and diagonalization of the Hamiltonian, we construct the supersymmetric generators of the system and their supersymmetric Lie algebra properties. We use a supersymmetric unitary transformation and diagonalize the Hamiltonian of the system. In section Time evolution wave functions and eigenvalues, we obtain the corresponding wave functions in two cases of the initial positions of both the atom and the field mode. In section Quantum aspects, we use the obtained wave functions in one of the two cases mentioned above and study the evolution of both the quasi-probability distribution *Q*-function and the atomic population inversion. We present and discuss their graphical results for three special cases of the level number *N*. We investigate the influence of the detuning and the Kerr medium on these evolutions. We end by section Discussion and conclusion, which concerns discussion and conclusion.

## Description of the model

**Fig. 1:**
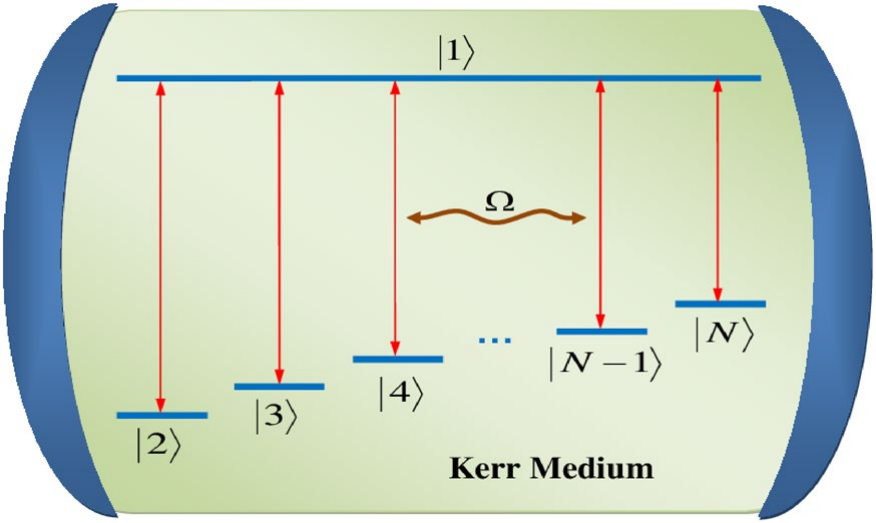
Schematic diagram of the interaction of *N*-level atom with one-mode cavity field in the presence of Kerr medium and multi-photon transitions.

The matter-field atomic system under consideration, which is an *N*-level atom interacting with one-mode field with frequency $\Omega$ in the presence of Kerr medium and multi-photon transitions, is represented in Fig. 1. This system can be visualized as an *N*-level atom within the framework of $(N-1)$ JCM. The top (excited) level is $|1\rangle$ and the lower (ground) levels are $|2\rangle$, $|3\rangle$, ..., $|N\rangle$ which assumed to be closely-separated. The transitions of $|1\rangle$ with the other $(N-1)$ levels are allowed but the transitions between the intermediate lower levels are forbidden. The Hamiltonian of this system can be written as ($\hbar =1$)

1 $$\begin{aligned} \hat{H}\,=\,\Omega \,\hat{a}^{\dagger } \hat{a}\,+\, \omega \,\hat{\sigma }_z\,+\,\chi \,\hat{a}^{\dagger 2} \hat{a}^{2} \,+\,\sqrt{N-1}\,g\, \Bigl (\hat{a}^k\,\hat{\sigma }_+ \,+\,\hat{a}^{\dagger k}\,\hat{\sigma }_- \Bigr ), \end{aligned}$$

where *k* is the photon number in the atom transition process, *g* is the coupling constant between photons and the atom, $\omega$ is the atom transition frequency, $\hat{a}^\dagger \, (\hat{a})$ is the creation (annihilation) operator of the field with the commutation relation $[\hat{a},\hat{a}^\dagger ]=1$, $\chi$ is the strength of the quadratic nonlinearity modelling the Kerr medium, and the raising (lowering) $\hat{\sigma }_+$ ($\hat{\sigma }_-$) and population $\hat{\sigma }_z$ operators of the atom are given by the $N\times N$ matrices

2 $$\begin{aligned} \hat{\sigma }_+\,=\,\frac{1}{\sqrt{N-1}}\,|1\rangle \Bigl (\sum _{j=2}^N \langle j|\Bigr )\,=\,\frac{1}{\sqrt{N-1}}\, \left( \begin{array}{cccccc} 0 & 1 & 1 & \cdots & 1\\ 0 & 0 & 0 & \cdots & 0\\ 0 & 0 & 0 & \cdots & 0\\ \vdots & \vdots & \vdots & \vdots & \vdots \\ 0 & 0 & 0 & \cdots & 0\\ \end{array} \right) , \end{aligned}$$

3 $$\begin{aligned} \hat{\sigma }_-\,=\,\frac{1}{\sqrt{N-1}}\,\Bigl (\sum _{j=2}^N |j\rangle \Bigr ) \langle 1|\,=\,\frac{1}{\sqrt{N-1}}\, \left( \begin{array}{cccccc} 0 & 0 & 0 & \cdots & 0\\ 1 & 0 & 0 & \cdots & 0\\ 1 & 0 & 0 & \cdots & 0\\ \vdots & \vdots & \vdots & \vdots & \vdots \\ 1 & 0 & 0 & \cdots & 0\\ \end{array} \right) , \end{aligned}$$

4 $$\begin{aligned} \begin{array}{cccc} \hat{\sigma }_z\,=\,\frac{1}{2(N-1)}\,\Bigl [(N-1) |1\rangle \langle 1|\,-\,\Bigl (\sum _{j=2}^N |j\rangle \Bigr )\Bigl (\sum _{j=2}^N \langle j|\Bigr ) \Bigr ]\\ \,=\,\frac{1}{2(N-1)} \left( \begin{array}{cccccc} (N-1) & 0 & 0 & \cdots & 0\\ 0 & -1 & -1 & \cdots & -1\\ 0 & -1 & -1 & \cdots & -1\\ \vdots & \vdots & \vdots & \vdots & \vdots \\ 0 & -1 & -1 & \cdots & -1\\ \end{array} \right) .\quad \end{array} \end{aligned}$$

It is easy to see that these matrices satisfy the *SU*(2) algebra

5 $$\begin{aligned} [\hat{\sigma }_+, \hat{\sigma }_-]\,=\,2\hat{\sigma }_z, \qquad \qquad [\hat{\sigma }_z, \hat{\sigma }_\pm ]\,=\,\pm \hat{\sigma }_\pm . \end{aligned}$$

## Supersymmetric generators and diagonalization of the Hamiltonian

To define the supersymmetric generators of the considered atomic system, we introduce the following $N\times N$ matrix operators

6 $$\begin{aligned} \hat{Q}^\dagger \,=\, \hat{a}^k\hat{\sigma }_+, \qquad \qquad \hat{Q}\,=\, \hat{a}^{\dagger k}\hat{\sigma }_-, \end{aligned}$$

then we construct the $N\times N$ matrix operator defined by the anti-commutation relation

7 $$\begin{aligned} \hat{N}'\,=\, \{\hat{Q}^\dagger , \hat{Q}\}\,=\, \frac{1}{(N-1)} \left( \begin{array}{cccccc} (N-1)\hat{a}^k\hat{a}^{\dagger k} & 0 & 0 & \cdots & 0\\ 0 & \hat{a}^{\dagger k}\hat{a}^{k} & \hat{a}^{\dagger k}\hat{a}^{k} & \cdots & \hat{a}^{\dagger k}\hat{a}^{k}\\ 0 & \hat{a}^{\dagger k}\hat{a}^{k} & \hat{a}^{\dagger k}\hat{a}^{k} & \cdots & \hat{a}^{\dagger k}\hat{a}^{k}\\ \vdots & \vdots & \vdots & \vdots & \vdots \\ 0 & \hat{a}^{\dagger k}\hat{a}^{k} & \hat{a}^{\dagger k}\hat{a}^{k} & \cdots & \hat{a}^{\dagger k}\hat{a}^{k}\\ \end{array} \right) . \end{aligned}$$

Clearly, the matrices $\hat{Q}^\dagger$, $\hat{Q}$ and $\hat{N}'$ form the supersymmetric generators and, as it is easy to prove, they satisfy the following supersymmetric Lie algebra properties

8 $$\begin{aligned} \begin{array}{cccccccc} [\hat{N}',\hat{n}]\,=\, [\hat{\sigma }_z,\hat{n}]\,=\,0,\qquad \qquad [\hat{N}',\hat{Q}]\,=\,[\hat{N}',\hat{Q}^\dagger ]\,=\,[\hat{N}',\hat{\sigma }_z]\,=\,0,\,\,\\ {[}\hat{Q},\hat{n}]\,=\, -k\hat{Q},\qquad \qquad \qquad \quad [\hat{Q}^\dagger , \hat{n}]\,=\,k\hat{Q}^\dagger ,\qquad \qquad \qquad \qquad \quad \\ \,[\hat{Q},\hat{\sigma }_z]\,=\,\hat{Q},\qquad \qquad \qquad \quad \,\,\,\,\, [\hat{Q}^\dagger , \hat{\sigma }_z]\,=\,-\hat{Q}^\dagger ,\,\,\,\qquad \qquad \qquad \qquad \\ {[}[\hat{Q}^\dagger , \hat{Q}] \,=\,2\hat{N}'\hat{\sigma }_z,\qquad \qquad \quad \,\,\,\, \hat{Q}^2\,=\,\hat{Q}^{\dagger 2}\,=\,0,\qquad \qquad \qquad \qquad \,\,\,\,\\ (\hat{Q}^\dagger - \hat{Q})^2\,=\,-\hat{N}'\qquad \qquad \quad \,\, \{\hat{\sigma }_z,\hat{Q}\}\,=\,\{\hat{\sigma }_z,\hat{Q}^\dagger \}\,=\,0,\quad \qquad \qquad \\ \hat{\sigma }_z(\hat{Q}^\dagger - \hat{Q})\,=\,\frac{1}{2}(\hat{Q}^\dagger + \hat{Q}),\qquad \qquad \qquad \qquad \qquad \qquad \qquad \qquad \quad \,\,\, \end{array} \end{aligned}$$

where $\hat{n}=\hat{a}^\dagger \hat{a}$ is the mean photon number operator. The Hamiltonian (1) is now becomes

9 $$\begin{aligned} \hat{H}\,=\,\Omega \, \hat{n}\,+\, \omega \,\hat{\sigma }_z\,+\,\chi \,\hat{n}(\hat{n}-1) \,+\,\sqrt{N-1}\,g\,(\hat{Q}^\dagger \,+\,\hat{Q}). \end{aligned}$$

In order to diagonalize the Hamiltonian (9), we introduce the supersymmetric unitary operator

10 $$\begin{aligned} \hat{T}\,=\,\hbox {exp} \Bigl ( -\frac{\hat{\theta }}{2}\,\frac{1}{\sqrt{\hat{N}'}}(\hat{Q}^\dagger -\hat{Q})\Bigr ). \end{aligned}$$

Using the properties (8), this operator can be rewritten as

11 $$\begin{aligned} \hat{T}\,=\,\hbox {cos}\,\frac{\hat{\theta }}{2}\,-\, \hbox {sin}\,\frac{\hat{\theta }}{2}\, \frac{1}{\sqrt{\hat{N}'}} (\hat{Q}^\dagger - \hat{Q}), \end{aligned}$$

with

12 $$\begin{aligned} \hat{T}^{-1}\,=\,\hbox {cos}\,\frac{\hat{\theta }}{2}\,+\, \hbox {sin}\,\frac{\hat{\theta }}{2}\, \frac{1}{\sqrt{\hat{N}'}} (\hat{Q}^\dagger - \hat{Q})\,=\,\hat{T}^\dagger . \end{aligned}$$

At this stage, we use equations (8), (11) and (12), and find that

13 $$\begin{aligned} \begin{array}{cccccccc} \,\qquad \hat{T}^{-1}\hat{n}\hat{T}\,=\,\hat{A}\,-\,k\, \hbox {cos}\,\hat{\theta }\,\hat{\sigma }_z\,+\,\frac{k}{2} \hbox {sin}\,\hat{\theta }\,\frac{1}{\sqrt{\hat{N}'}}\,(\hat{Q}^\dagger +\hat{Q}), \qquad \qquad \,\\ \,\,\,\,\quad \quad \hat{T}^{-1}\hat{\sigma }_z\hat{T}\,=\,\hbox {cos}\,\hat{\theta }\,\hat{\sigma }_z\,-\, \frac{1}{2}\,\hbox {sin}\,\hat{\theta }\,\frac{1}{\sqrt{\hat{N}'}}\,(\hat{Q}^\dagger +\hat{Q}), \qquad \qquad \qquad \qquad \\ \,\,\,\,\quad \hat{T}^{-1}\hat{n}(\hat{n}-1)\hat{T}\,=\, \hat{A}(\hat{A}-1)\,+\,k\,(1-2\hat{A})\hbox {cos}\,\hat{\theta }\,\hat{\sigma }_z \,+\,k^2 \hat{\sigma }_z^2 \qquad \qquad \qquad \quad \\ \,-\,\frac{k}{2} \hbox {sin}\,\hat{\theta } \,\frac{1}{\sqrt{\hat{N}'}}\,(1-2\hat{A})(\hat{Q}^\dagger +\hat{Q}),\\ \hat{T}^{-1}(\hat{Q}^\dagger +\hat{Q})\hat{T}\,=\,\hbox {cos}\,\hat{\theta }\, (\hat{Q}^\dagger +\hat{Q})\,+\,2\,\hbox {sin}\,\hat{\theta }\,\sqrt{\hat{N}'}\,\hat{\sigma }_z, \qquad \qquad \qquad \qquad \,\,\\ \end{array} \end{aligned}$$

with the operator

14 $$\begin{aligned} \hat{A}\,=\,\hat{n}\,+\,k\,\hat{\sigma }_z. \end{aligned}$$

The diagonalization of the Hamiltonian (9) can now be performed and obtain

15 $$\begin{aligned} \begin{array}{cccc} \hat{H}'\,=\,\hat{T}^{-1} \hat{H}\,\hat{T}\qquad \qquad \qquad \qquad \qquad \qquad \qquad \qquad \qquad \qquad \qquad \qquad \qquad \qquad \qquad \\ \,=\, \Omega \,\hat{A}\,+\,\chi \,\hat{A}(\hat{A}-1)\,-\,\hat{B}\, \hbox {cos}\,\hat{\theta }\,\hat{\sigma }_z\,+\, k^2\chi \, \hat{\sigma }_z^2\qquad \qquad \qquad \qquad \qquad \qquad \qquad \\ +\,2\sqrt{N-1}\,g\,\hbox {sin}\,\hat{\theta }\,\sqrt{\hat{N}'}\,\hat{\sigma }_z \,+\,\Bigl [\frac{\hat{B}}{2\sqrt{\hat{N}'}}\,\hbox {sin}\,\hat{\theta } \,+\,\sqrt{N-1}\,g\,\hbox {cos}\,\hat{\theta }\Bigr ](\hat{Q}^\dagger +\hat{Q}), \end{array} \end{aligned}$$

where the operator $\hat{B}$ is given by

16 $$\begin{aligned} \hat{B}\,=\,\Delta \,-\,k\,\chi (1-2\hat{A}), \end{aligned}$$

with the detuning parameter $\Delta =k\Omega -\omega$. Choosing $\hat{\theta }$ such that $\hbox {tan}\,\hat{\theta } = -2\,g\,\sqrt{(N-1)\hat{N}'}/\hat{B}$, we obtain the diagonalized Hamiltonian in the form:

17 $$\begin{aligned} \hat{H}'\,=\,\Omega \,\hat{A}\,+\,\chi \,\hat{A}(\hat{A}-1)\,+\, k^2\chi \,\hat{\sigma }_z^2 \,-\,\sqrt{\hat{B}^2+4(N-1)g^2\hat{N}'}\,\hat{\sigma }_z. \end{aligned}$$

Next, we shall go forward and construct the time evolution atom-field wave function of the considered atomic system.

## Time evolution wave functions and eigenvalues

Having diagonalized the Hamiltonian of the considered atomic system, we introduce the following orthonormal eigenstates of $\hat{H}'$

18 $$\begin{aligned} |\psi '_+\rangle \,=\, \left( \begin{array}{cccccc} |n\rangle \\ 0 \\ 0 \\ \vdots \\ 0 \\ \end{array} \right) ,\qquad \quad |\psi '_-\rangle \,=\,\frac{1}{\sqrt{N-1}}\, \left( \begin{array}{cccccc} 0 \\ |n+k\rangle \\ |n+k\rangle \\ \vdots \\ |n+k\rangle \\ \end{array} \right) , \end{aligned}$$

where $|n\rangle$ is the Fock-state of the field. These *N*-vector eigenstates satisfy the following relations

19 $$\begin{aligned} \begin{array}{cccc} \hat{\sigma }_z|\psi '_\pm \rangle \,=\,\pm \frac{1}{2}|\psi '_\pm \rangle ,\qquad \qquad \hat{A}|\psi '_\pm \rangle \,=\,(n+\frac{k}{2}) |\psi '_\pm \rangle ,\qquad \\ \,\hat{B}\,|\psi '_\pm \rangle \,=\,2\alpha _1|\psi '_\pm \rangle ,\qquad \,\,\quad \hat{N}'|\psi '_\pm \rangle \,=\,\beta _1|\psi '_\pm \rangle ,\qquad \qquad \,\, \end{array} \end{aligned}$$

with

20 $$\begin{aligned} \alpha _1\,=\,\frac{1}{2}(\Delta - k\chi )\,+\,k\chi (n+\frac{k}{2}), \qquad \qquad \beta _1\,=\,\frac{(n+k)!}{n!}. \end{aligned}$$

At this stage, the operation of the diagonalized Hamiltonian on the eigenstates $|\psi '_\pm \rangle$ implies the following eigenvalues of the considered atomic system

21 $$\begin{aligned} F_\pm \,=\,\xi \,\mp \,\eta , \end{aligned}$$

with

22 $$\begin{aligned} \xi \,=\,\Omega (n+\frac{k}{2})\,+\,\chi (n+\frac{k}{2})(n-1+\frac{k}{2})\,+\,\frac{1}{4}k^2\chi ,\qquad \qquad \eta \,=\,\sqrt{\alpha _1^2+(N-1)g^2\beta _1}. \end{aligned}$$

The corresponding eigenstates can be constructed as

23 $$\begin{aligned} \begin{array}{cccc} |\psi _+\rangle \,=\,\hat{T}\, |\psi '_+\rangle \,=\,\hbox {cos}\,\frac{\hat{\theta }}{2}\,|\psi '_+\rangle \,+\,\hbox {sin}\,\frac{\hat{\theta }}{2}\,|\psi '_-\rangle ,\\ |\psi _-\rangle \,=\,\hat{T}\, |\psi '_-\rangle \,=\,\hbox {cos}\,\frac{\hat{\theta }}{2}\,|\psi '_-\rangle \,-\,\hbox {sin}\,\frac{\hat{\theta }}{2}\,|\psi '_+\rangle . \end{array} \end{aligned}$$

Here we have to point out that the Hamiltonian $\hat{H}$ has another eigenstate given by the *N*-vector

24 $$\begin{aligned} |\psi \rangle \,=\,\frac{1}{\sqrt{N-1}}\, \left( \begin{array}{cccccc} 0 \\ |n\rangle \\ |n\rangle \\ \vdots \\ |n\rangle \\ \end{array} \right) ,\qquad \quad n\le k-1 \end{aligned}$$

which is not included in (23). This eigenstate satisfies the relations

25 $$\begin{aligned} \begin{array}{cccc} \hat{\sigma }_z\,|\psi \rangle \,=\,-\frac{1}{2}\,|\psi \rangle ,\\ \hat{n}\,|\psi \rangle \,=\,n\,|\psi \rangle ,\,\,\\ (\hat{Q}^\dagger +\hat{Q})\,|\psi \rangle \,=\,0.\qquad \qquad \quad \,\, \end{array} \end{aligned}$$

Therefore, the eigenvalue *F* corresponding to $|\psi \rangle$ can be easily obtained and find that

26 $$\begin{aligned} F\,=\,\Omega (n-\frac{k}{2})\,+\,\frac{\Delta }{2}\,+\,\chi n(n-1). \end{aligned}$$

Having constructed the eigenstates $|\psi _\pm \rangle$ and $|\psi \rangle$ with the corresponding eigenvalues $F_\pm$ and *F*, we can now construct the time evolution wave function $|\psi (t)\rangle$ of the considered atomic system. We assume that the initial state of the system is the arbitrary state $|\psi (0)\rangle$. Therefore, the solution of the Schrödinger equation is

27 $$\begin{aligned} |\psi (t)\rangle \,=\,e^{-i\hat{H}t}\,|\psi (0)\rangle . \end{aligned}$$

From the completeness condition of the whole space and using equations (18), (21)-(24), and (26), the wave function $|\psi (t)\rangle$ can be obtained and find that

28 $$\begin{aligned}&|\psi (t)\rangle \,=\,\sum _{n=0}^\infty \,e^{-i\xi t}\Bigl [ \Bigl ( \alpha _n\,\hbox {cos}\,\frac{\hat{\theta }}{2}\,e^{i\eta t}\,-\, \beta _n\,\hbox {sin}\,\frac{\hat{\theta }}{2}\,e^{-i\eta t}\Bigr )|\psi '_+ \rangle \nonumber \\&\qquad \qquad \,+\,\Bigl ( \alpha _n\,\hbox {sin}\,\frac{\hat{\theta }}{2}\,e^{i\eta t}\,+\, \beta _n\,\hbox {cos}\,\frac{\hat{\theta }}{2}\,e^{-i\eta t}\Bigr )|\psi '_- \rangle \Bigr ]\,+\,\sum _{n=0}^{(k-1)}\,\gamma _n\,e^{-iFt}|\psi \rangle , \end{aligned}$$

where

29 $$\begin{aligned} \alpha _n\,=\,\langle \psi _+|\psi (0)\rangle ,\qquad \beta _n\,=\,\langle \psi _-|\psi (0)\rangle ,\qquad \gamma _n\,=\,\langle \psi |\psi (0)\rangle . \end{aligned}$$

Let us consider the following two different cases of the initial positions of the atom and the field mode.

(I): The initial position of the atom is in the state $|\psi '_+ \rangle$ and that of the field mode is in a coherent state $|\alpha \rangle$, that is
  30 $$\begin{aligned} |\psi (0)\rangle \,=\,q_n\,|\psi '_+ \rangle , \end{aligned}$$
  where $q_n\,=\,e^{-\bar{n}/2}\,\alpha ^n/\sqrt{n!}$, with the initial mean photon number $\bar{n}=|\alpha |^2$. Thus we find that
  31 $$\begin{aligned} \alpha _n\,=\,q_n\,\hbox {cos}\,\frac{\hat{\theta }}{2},\qquad \beta _n\,=\,-q_n\,\hbox {sin}\,\frac{\hat{\theta }}{2},\qquad \gamma _n\,=\,0. \end{aligned}$$
  In this case, the atom-field wave function (28) becomes
  32 $$\begin{aligned} |\psi (t)\rangle \,=\,\sum _{n=0}^\infty \,q_n\,\Bigl ( A(t) |\psi '_+\rangle \,+\,B(t) |\psi '_-\rangle \Bigr ), \end{aligned}$$
  where
  33 $$\begin{aligned} \begin{array}{cccc} A(t)\,=\,\Bigl (\hbox {cos}\,\eta t \,+\,i\alpha _1\, \frac{\hbox {sin}\,\eta t}{\eta }\Bigr )e^{-i\xi t},\,\,\,\\ B(t)\,=\,-ig\sqrt{(N-1)\beta _1}\,\frac{\hbox {sin}\,\eta t}{\eta }e^{-i\xi t}. \end{array} \end{aligned}$$

(II): The atom is initially in the state $|\psi '_- \rangle$ and the field mode is initially in a coherent state $|\alpha \rangle$, that is
  34 $$\begin{aligned} |\psi (0)\rangle \,=\,q_{n+k}\,|\psi '_- \rangle , \end{aligned}$$
  where $q_{n+k}\,=\,e^{-\bar{n}/2}\,\alpha ^{(n+k)}/\sqrt{(n+k)!}$. Thus we find that
  35 $$\begin{aligned} \alpha _n\,=\,q_{n+k}\,\hbox {sin}\,\frac{\hat{\theta }}{2},\qquad \beta _n\,=\,q_{n+k}\,\hbox {cos}\,\frac{\hat{\theta }}{2},\qquad \gamma _n\,=\,q_n. \end{aligned}$$
  In this case, the atom-field wave function (28) takes the form
  36 $$\begin{aligned} |\psi (t)\rangle \,=\,\sum _{n=0}^\infty \,q_{n+k}\,\Bigl ( C(t) |\psi '_+\rangle \,+\,D(t) |\psi '_-\rangle \Bigr )\,+\, \sum _{n=0}^{k-1}\,q_nE(t)|\psi \rangle , \end{aligned}$$
  where
  37 $$\begin{aligned} \begin{array}{cccc} C(t)\,=\,-ig\sqrt{(N-1)\beta _1}\,\frac{\hbox {sin}\,\eta t}{\eta }e^{-i\xi t},\\ D(t)\,=\,\Bigl (\hbox {cos}\,\eta t \,-\,i\alpha _1\, \frac{\hbox {sin}\,\eta t}{\eta }\Bigr )e^{-i\xi t},\,\,\\ E(t)\,=\,e^{-iFt}.\qquad \qquad \qquad \qquad \quad \end{array} \end{aligned}$$

At this stage, we can study any quantum aspects of the considered atomic system. This is what we shall do in the next section.

## Quantum aspects

Now, we are ready to provide analytical formulas for any physical quantity using the obtained time dependent wave function of the considered atomic system. In particular, we shall investigate two main quantum aspects, namely, the quasi-probability distribution *Q*-function and the atomic population inversion. In terms of the wave functions obtained before, the reduced density matrix of the field is defined by $\hat{\rho }_f(t)=|\psi (t)\rangle \langle \psi (t)|$. We shall restrict our investigations on the first case of the initial positions of the atom and the field mode and therefore, we shall use the atom-field wave function given by (32) and (33).

### Quasi-probability distribution *Q*-function

One of the most important quantum aspects of the field is to investigate the evolution of the quasi-probability distribution *Q*-function. This function has found a lot of applications in the field of quantum optics. It provides a complete information about the atom-field interaction system^32^, it is always nonnegative, bounded, exists for any density matrix, and has no singularity problems at all. Moreover, this function can be detected in homodyne experiments^33,34^. Also, the width of this function is used as a measure for field squeezing.

**Fig. 2:**
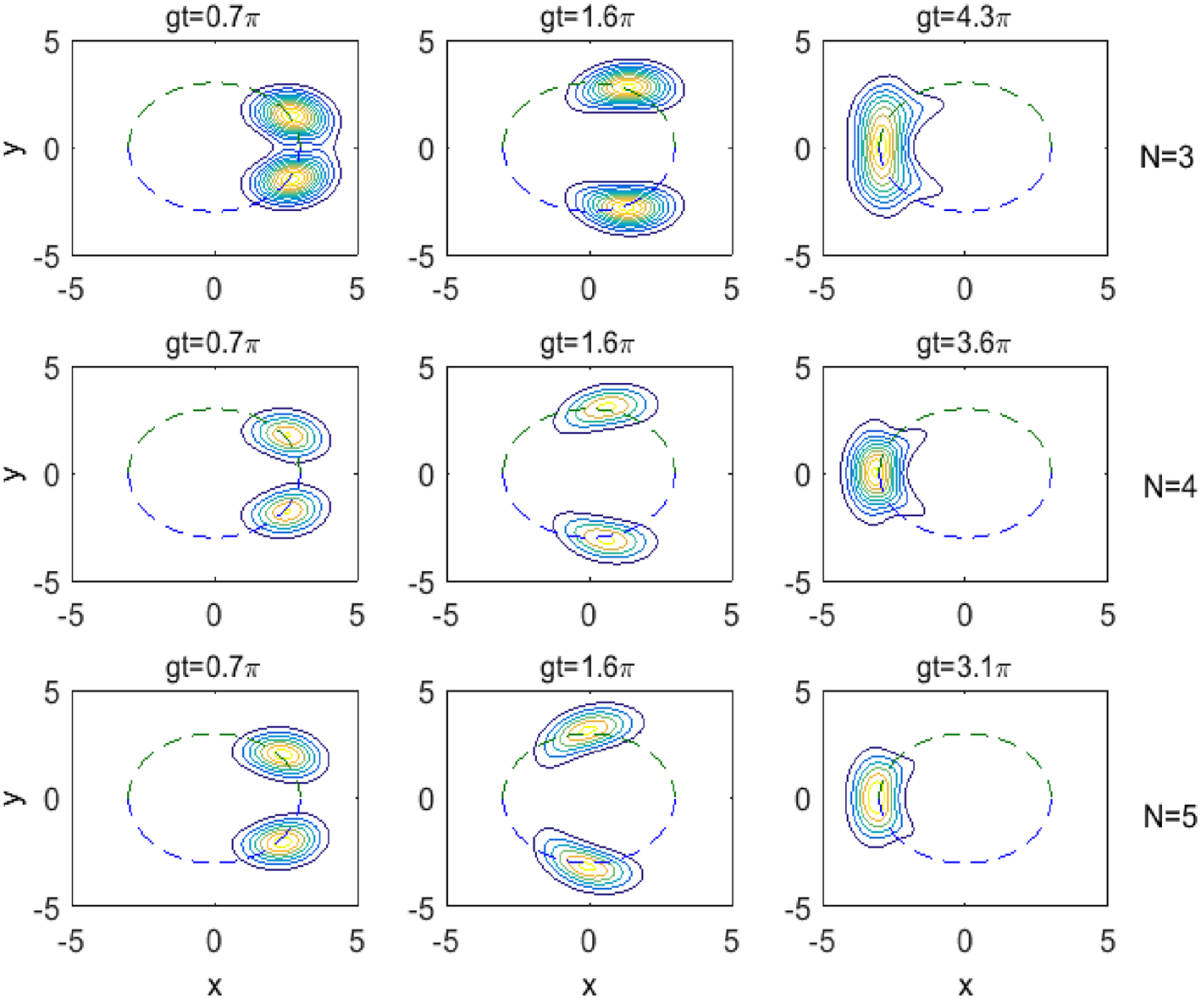
The contour diagram of the *Q*-function versus *x* (horizontal axes) and *y* (vertical axes) for $\Delta =\chi =0.0$, $\bar{n}=9$, $k=1$, $N=3,\,4,\,5$ and various values of the scaled time *gt* as given on each subplot.

Using the reduced field density operator, the quasi-probability distribution *Q*-function can be expressed as the coherent state expectation value of the operator in the form

38 $$\begin{aligned} Q(\alpha ,t)\,=\,\frac{1}{\pi }\,\langle \alpha | \hat{\rho }_f |\alpha \rangle , \end{aligned}$$

where $|\alpha \rangle$ is the coherent state of the field defined by

39 $$\begin{aligned} |\alpha \rangle \,=\,\sum _{n=0}^\infty \,e^{-\frac{\alpha ^2}{2}} \frac{\alpha ^n}{\sqrt{n!}}\,|n\rangle . \end{aligned}$$

In our considered atomic system, we use equations (32) and (33) and find that

40 $$\begin{aligned} Q(\alpha ,t)\,=\,\frac{1}{\pi }\,\Bigl [ |\langle \alpha |A(t)\rangle |^2\,+\,|\langle \alpha |B(t)\rangle |^2\Bigr ], \end{aligned}$$

with

41 $$\begin{aligned} \langle \alpha |A(t)\rangle \,=\,\sum _{n=0}^\infty \,q_n e^{-\frac{\alpha ^2}{2}} \frac{\alpha ^{*n}}{\sqrt{n!}}\,A(t), \end{aligned}$$

and

42 $$\begin{aligned} \langle \alpha |B(t)\rangle \,=\,\sqrt{N-1}\sum _{n=0}^\infty \,q_n e^{-\frac{\alpha ^2}{2}} \frac{\alpha ^{*(n+k)}}{\sqrt{(n+k)!}}\,B(t). \end{aligned}$$

Fig. 2 shows the contour plots of the quasi-probability distribution *Q*-function given by (40)-(42) in the complex $\alpha$-plane ($\alpha =x+iy$). In this figure, we consider the transition processes $k=1$, the mean photon number $\bar{n}=9$, various values of the scaled time *gt* as given on each subplot. Also, the resonance case ($\Delta =0.0$) and the absence of the Kerr medium ($\chi =0.0$) are considered in this figure. Moreover, we have considered three special cases of the level number *N*, where the first row of the subplots corresponds to $N=3$, the second row for $N=4$, and the third row corresponds to $N=5$.

In view of Fig. 2, we observe that the *Q*-function represented in the beginning ($gt=0.0$) by one peak at the right of the grid, and the center of the peak lies on a circle with radius $\sqrt{\bar{n}}$ centered at $x=y=0$. As soon as the time goes up, splitting occurs in this peak into two peaks each rotates on the mentioned circle opposite to each other until they collide and pass through each other at the left side of the grid. Then they repeat rotation each in its direction until they collide and pass through each other again at the original position on the right of the grid and so on. We observe also that there is broadening occurs in the distribution of *Q*-function, which increases with increasing the time.

The effect of the level number *N* on the evolution of *Q*-function can be observed in Fig.2 as well, where we see that as *N* increases, the splitting and the rotation become faster. This is clear as shown from the subplots, where for example, if we look at the first column of Fig. 2, we find that the peak in the beginning starts splitting into two peaks and when the scaled time $gt=0.7\pi$ it takes the upper shape of the first column for $N=3$ and takes the middle shape for $N=4$ and the bottom shape for $N=5$. Clearly, the splitting and rotation happen faster as the number of levels increases. Also, from the third column of Fig. 2, where the two splitting peaks collide and pass through each other for the first time, we find that the first collision for $N=3$ occurs when $gt=4.3\pi$ and this time reduces to $3.6\pi$ for $N=4$ and to $3.1\pi$ for $N=5$. This indicates that as *N* increases, the rotation of the two peaks becomes faster.

**Fig. 3:**
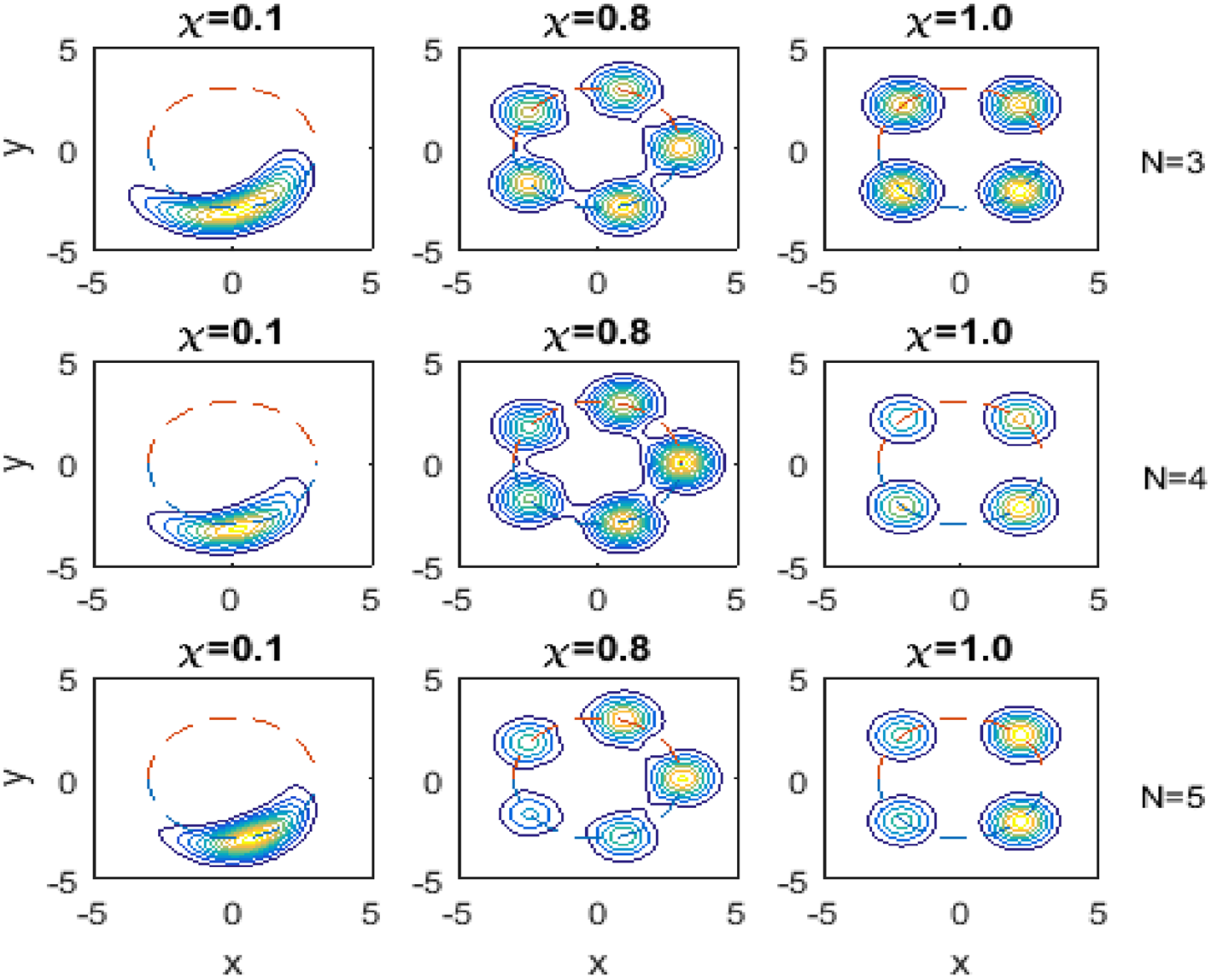
The same as in Fig. 2 but for fixed value of the scaled time $gt =\pi /4$, and different values of $\chi$ as given on each subplot.

To visualize the influence of the Kerr medium on the evolution of the quasi-probability distribution *Q*-function, we sketch the contour plots of this function in Fig. 3. In this figure, we consider the same situations as in Fig. 2 but for different values of the Kerr medium parameter $\chi$ as shown on each subplot, and for fixed value of the scaled time $gt =\frac{\pi }{4}$.

We observe from Fig. 3 that as soon as the Kerr medium takes place, the peak of the distribution of *Q*-function at the beginning rotates clockwise on the circle mentioned before and there is no splitting but broadening occurs in the peak. This broadening increases with increasing the Kerr medium parameter $\chi$ resulting in a banana-like shape (see the first column of the subplots). Increasing $\chi$ a bit more, the banana-like shape becomes ring-like shape with multi-peaks (see the second column of the subplots). As $\chi \longrightarrow 1$, the ring-like shape starts breaking with less and less separate peaks ending with only four peaks at $\chi =1$ as shown in the third column of the subplots. It should be reported here that if we fixed the scaled time to the value $gt=\frac{\pi }{\ell }$, for integer $\ell$, we will have $\ell$ distinct peaks at $\chi =1$.

The increment of the level number *N* on the distribution of *Q*-function can be observed in Fig. 3 as well, where as *N* increases the rotation of the peak and accordingly the broadening becomes faster. This can be observed from the second column of the subplots of Fig. 3 which indicates that for the fixed value $\chi =0.8$, the ring-like shape with multi-peaks starts splitting faster as *N* increases. Moreover, the third column of the subplots of Fig. 3, where we take the fixed value $\chi =1$, the multi-peaks in the ring-like shape start breaking with less and less separate peaks ending with only four peaks at $\chi =1$ (where we have taken a fixed value of the scaled time $gt =\pi /4$) for any number of levels *N*.

**Fig. 4:**
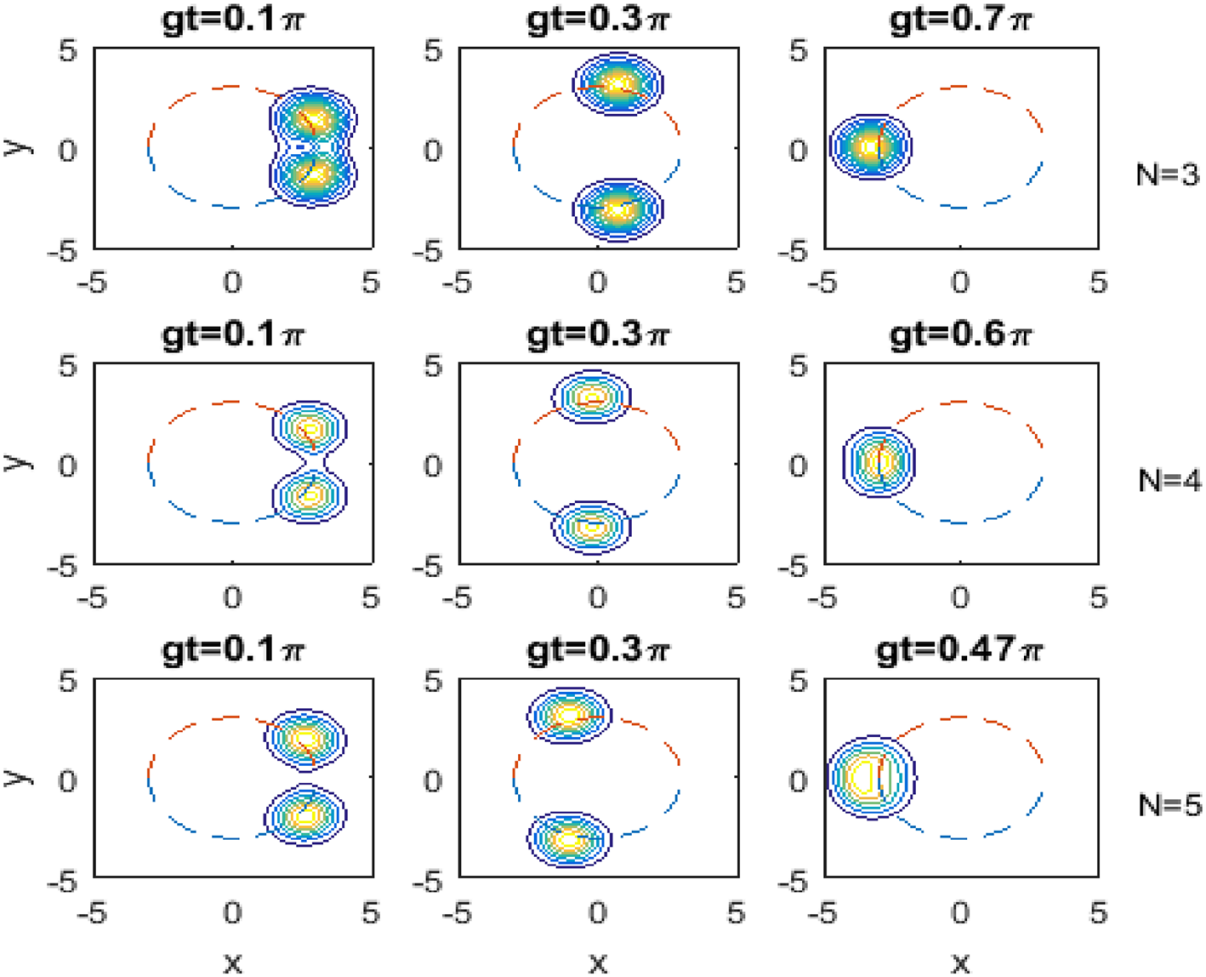
The same as in Fig. 2 but for $k=2$ and various values of the scaled time *gt* as given on each subplot.

Fig. 4 shows the contour plots of the evolution of the quasi-probability distribution *Q*-function for the same situations as in Fig. 2 but for the transition processes $k=2$, and various values of the scaled time *gt* as indicated on each subplot.

In view of Fig. 4, we observe that the behavior of the evolution of the quasi-probability distribution *Q*-function in the transition processes $k=2$ is exactly the same as in the transition processes $k=1$ discussed in Fig. 2 except that firstly there is no broadening occurs in the peaks, and secondly the splitting and rotation of the peaks are more faster in the $k=2$ than in $k=1$ case.

### Atomic population inversion

The atomic population inversion is one of the most important quantum aspects in the context of atom-field interactions. It describes the state of the atom during the time evolution. It is defined as the difference in the probabilities of finding the atom in the excited states and in the ground states. It has been reported for many schemes of different systems^35–37^. It is well known that the atomic population inversion is given by the expectation value of the atomic inversion operator $\hat{\sigma }_z$^35,36,37,38^. Therefore, the atomic population inversion is the simplest important quantity to be calculated.

For the considered atomic system, we use $\hat{\sigma }_z$ given by equation (4) and use the wave function given by equations (32) and (33) and find that

43 $$\begin{aligned} \langle \hat{\sigma }_z(t)\rangle \,=\,\langle \psi (t)| \hat{\sigma }_z |\psi (t)\rangle \,=\,\frac{1}{2}\,\sum _{n=0}^\infty \,P_n\,\Bigl (|A(t)|^2\,-\,|B(t)|^2\Bigr ), \end{aligned}$$

where $P_n= |q_n|^2$ is the initial photon distribution of the field mode.

**Fig. 5:**
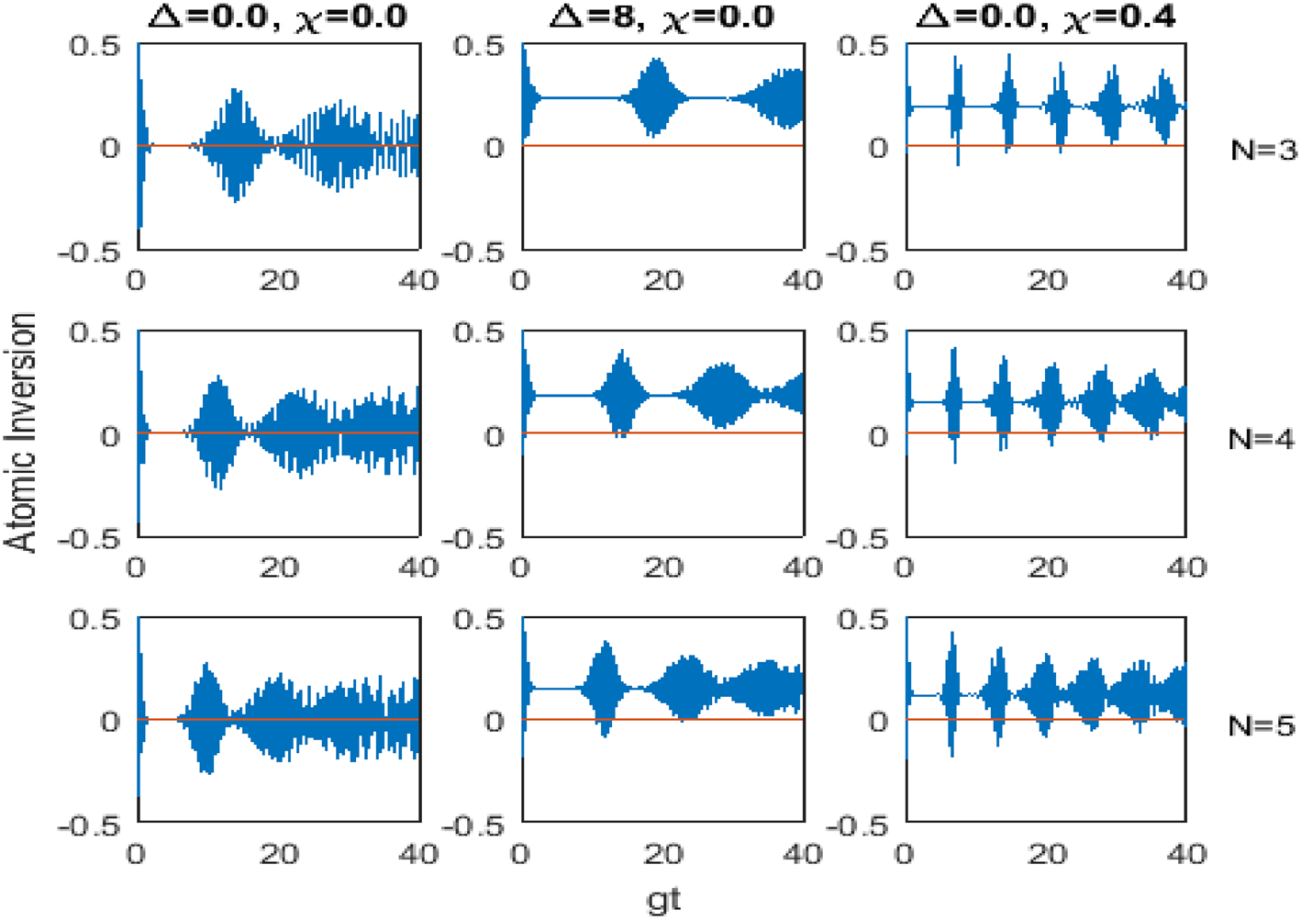
The atomic inversion (vertical axes) versus the scaled time *gt* (horizontal axes) for $k=1$, $\bar{n}=9$, different values of the parameters $\Delta$ and $\chi$ ($\Delta = 0.0$, $\chi = 0.0$ for $1^{\hbox {st}}$ column of the subplots, $\Delta = 8$, $\chi = 0.0$ for $2^{\hbox {nd}}$ column, and $\Delta = 0.0$, $\chi = 0.4$ for $3^{\hbox {rd}}$ column), and for three special cases of the level number *N* ($N=3$ for $1^{\hbox {st}}$ row of the subplots, $N=4$ for $2^{\hbox {nd}}$ row and $N=5$ for $3^{\hbox {rd}}$ row).

The time evolution of the atomic inversion (42) can be shown in Fig. 5, where we plotted $\langle \hat{\sigma }_z(t)\rangle$ against the scaled time *gt* for the transition processes $k=1$, the mean photon number $\bar{n}=9$, and for three special cases of the level number $N=3$ represented by the first row of the subplots, $N=4$ represented by the second row of the subplots, and $N=5$ represented by the third row of the subplots. The first column of the subplots of this figure shows the time evolution of the atomic inversion in the resonance case $(\Delta =0.0)$ and the absence of the Kerr medium $(\chi =0.0)$. The second column of the subplots concerns the non-resonance where we see the influence of the detuning parameter $(\Delta =8)$ in the absence of the Kerr medium. The third column of the subplots of Fig. 5 shows the effect of the Kerr medium parameter $(\chi =0.4)$ on the evolution of the atomic inversion in the resonance case.

Clearly, we observe the phenomenon of collapses and revivals in Fig. 5 for the time evolution of the atomic inversion. As shown in the first column of the subplots, the atomic inversion varies between positive and negative values. However, the influence of the detuning or the Kerr medium shown in the second and third columns of the subplots, respectively, directed the evolution to the positive side, which is physically means that more energy is stored inside the atomic system. Moreover, the increment of the level number *N* reduces the collapse time as well as reduces the storage of energy inside the atomic system.

## Discussion and conclusion

This article studies a generalized atomic system of *N*-level atom within the framework of ($N-1$) JCM interacting with a single-mode cavity field in the presence of Kerr medium and multi-photon transition. This system includes the following special cases of atomic systems: the three-level $\Lambda$-configuration system when $N=3$, the four-level *m*-configuration system when $N=4$, and the five-level double $\Lambda$-configuration system when $N=5$. We showed that this system possesses supersymmetric structure. The analytic solution of this generalized system is obtained via the supersymmetric unitary transformation method. The corresponding supersymmetric generators are constructed and the Hamiltonian of the system is diagonalized. The atom-field wave functions are obtained and the quasi-probability distribution *Q*-function and the atomic population inversion are investigated as two main quantum aspects. The used method is a simple and straightforward analytic way to solve any atomic system once the corresponding supersymmetric generators are constructed. This method allows a unified treatment of a variety atom-field interaction systems and it can enrich the contents of supersymmetric quantum mechanics.

In addition to the universality and advantages of the used method, let us summarize the results of the investigations of the evolution of the two main quantum aspects mentioned above for the three special cases of atomic systems mentioned before.

$\bullet$ The atomic inversion exhibits collapse-revival phenomenon, which gives information about atom-field entanglement and disentanglement during the dynamics. This phenomenon is related to the evolution of the quasi-probability distribution *Q*-function, where collapses occur during the splitting of the distribution and revivals occur when the peaks of the distribution collide and pass through each other.

$\bullet$ The Kerr medium or the detuning parameter has an important effect on the evolution of the atomic inversion, where their effect shifts the evolution to the positive side. Physically, this means that more energy stored inside the atomic system.

$\bullet$ The increment of the level number *N* on the evolution of the atomic inversion reduces the collapse time as well as reduces the storage of energy inside the atomic system. In the other hand, this increment makes the splitting and rotation of the peaks of the distribution of *Q*-function more faster.

$\bullet$ The banana (ring) shapes occurred in the distribution of *Q*-function corresponds to the sub-Poissonian (super-Poissonian) states, and these shapes are due to the influence of the Kerr medium.

## Data Availability

The datasets used and/or analyzed during the current study are available from the corresponding author on reasonable request.
